# Composition of Household Income and Child Nutrition Outcomes Evidence from Uganda

**DOI:** 10.1016/j.worlddev.2017.03.023

**Published:** 2018-09

**Authors:** Angeli Kirk, Talip Kilic, Calogero Carletto

**Affiliations:** a University of California, Berkeley, USA; b The World Bank, Rome, Italy

**Keywords:** Child nutrition, Household income, Agriculture, Nutrition-sensitive agricultural production, Uganda, Sub-Saharan Africa

## Abstract

This study targets the empirical space between cross-country analyses exploring links between income and nutrition without insights on micro-level determinants, and relevant microeconomic studies hindered by small sample size and/or incomplete data. We use the rural samples of the three waves of the Uganda National Panel Survey, and estimate panel regressions of child height-for-age *z*-scores (HAZ) controlling for time-invariant child-level heterogeneity. On the whole, we find no impact of short-term changes in total gross income on HAZ but document small positive correlations for younger children. Sector-differentiated analyses indicate that compared to wage earnings, only share of income from non-farm self-employment correlates positively with HAZ. Within agriculture, shares of income from consumption of own crop production and from low-protein crop production underlie the negative effect of share of income from crop production. While we cannot claim causal relationships, our findings suggest the possibility of “stickiness” of crop production to own consumption.

## Highlights

• This study takes advantage of income and height measurements from three years of the Uganda National Panel Survey.
• No correlation of short-term changes in income and height measures overall but small positive correlation for younger kids.
• Compared to wages, only share of income from nonfarm self-employment correlates positively with HAZ in the sector analysis.
• In agriculture, income shares from (i) consumption of own crops and (ii) low-protein crop production show negative effects.
• Overall, any effects are small, in a context where many households maintain a diversified portfolio.

## 1 Introduction

In the quest for widespread and sustainable welfare gains, not all income may have equal effects. Growth within some sectors or accruing to certain individuals within a population may be relatively more effective at reducing poverty and improving specific welfare outcomes in developing countries. Child under-nutrition, targeted directly by the first of the Millennium Development Goals and related to others, is an aspect of poverty that is often argued to be sensitive to growth in the agricultural sector, with potential for both gains and losses. In recent years, there has been a growing movement to pull together evidence on the links among agriculture, income, nutrition, and health for the design of multi-sectoral interventions that target nutritional deficiencies.^1^

All income has the potential to benefit children’s nutrition, and if household consumption choices depend on production outcomes only via total earnings, income from any source or sector will be equally beneficial. Empirically observed deviations from this theoretical case may originate from multiple sources: distribution of poverty across sectors, relative food production and consumption prices due to markups and transaction costs, risk preferences, and intra-household bargaining outcomes, to name a few. If such deviations occur, the direction and relative weights of these channels of impact would lead to very different prescriptions for policymaking and allocation of scarce resources meant to boost nutrition-supporting growth. Empirically, however, validation of the claims regarding whether and how household sectoral involvement and gains in productivity can contribute to changes in nutritional status and health has been hindered by data limitations and by methodological concerns.

A large collection of microeconomic studies attempting to determine the income links to nutrition through specific mechanisms provide mixed and often conflicting results. The investigated mechanisms include (i) commercialization (reviewed by DeWalt, 1993, Kennedy et al., 1992, Von Braun and Kennedy, 1994), (ii) gender dynamics (reviewed by Kurz and Johnson-Welch, 2007, Peña et al., 1996, Quisumbing et al., 1995, Quisumbing and Maluccio, 2000), and (iii) nutrition-sensitive production and education interventions (reviewed by Berti et al., 2004, Gillespie and Mason, 1994, Leroy and Frongillo, 2007, Masset et al., 2011^2^; Ruel, 2001, Soleri et al., 1991). While some differences could be due to context-specific dynamics, numerous reviews in recent years express concerns regarding (i) the validity of the empirical methods used for impact estimation, and (ii) the inconsistency in the types of data used across studies which often lack information on income and have information on only consumption or anthropometry but not both (Arimond et al., 2011, Leroy et al., 2008, World Bank, 2007).

Despite these challenges, the sheer number of studies conducted over the last few decades speaks to the long-standing and urgent demand for insights into how to effectively leverage growth for nutritional improvement. While researchers and key policy players overwhelmingly assert that there is a strong potential for agricultural development to support nutrition and health, they also lament the lack of insight into the specific conditions necessary and sufficient to achieve improved nutritional outcomes efficiently and at broad scale. Herforth (2013) synthesizes the current state of knowledge cites general consensus on many best practices for improving nutrition through agriculture but highlights two questions that are yet to be settled: (i) what are the relative nutritional impacts of agricultural production for own consumption *vis-à-vis* agricultural production for sales and (ii) what agricultural products households should focus on, for example staple crops vs. animal-source foods? To this list, we add a third, overarching question that stems from the literature: Even if agricultural growth can be leveraged effectively for nutrition, is it more effective than non-agricultural growth at micro level?

With these questions in mind, we take advantage of the three waves of the household survey data from the Uganda National Panel Survey in an attempt to fill the knowledge gap between the cross-country analyses that explore the links between income and nutrition but cannot explore determinants at a micro level and the numerous smaller microeconomic studies that point to mechanisms of impact but are often hindered by some combination of sample size, data incompleteness, and other methodological considerations. We start by looking at how child nutritional outcomes correlate with *short-term* changes (1–2 years) in household income regardless of source. Subsequently, we explore heterogeneity by source of income, first between crop cultivation^3^ and non-crop sources and then further within type of agriculture, according to the priorities set previously in the literature.

There are three key findings. First, we document no detectable impact of short-term changes in total gross income on height-for-age overall, though there may be a very small gain for the youngest children. Second, sector-differentiated analyses indicate that only the share of income originating from self-employment exerts positive and statistically significant effects on height relative to other sectors. Third, the income shares pertaining to (i) a household’s consumption of own crop production and (ii) low-protein crop production, rather than crop production alone, appear to be driving the negative effect of the share of income originating from crop production. All of these relationships are small relative to typical year-on-year changes in income composition.

The remainder of the paper is structured as follows. Section 2 discusses the theoretical mechanisms through which income growth and sector and subsector of growth can influence nutrition in the context of the existing body literature. Sections 3 describes our data sources; Section 4, empirical strategy and results. Section 5 concludes.

## 2 Linking income and agriculture to nutrition: theory and literature

The factors that are commonly understood to interact to that hinder nutrition are (1) household food insecurity, which encompasses food availability as well as quality, (2) inadequate care, and (3) unhealthy environment (Behrman and Deolalikar, 1988, UNICEF, 1990).^4^ The direction of these biologically based impacts is well established in the literature, and we take them as given: any positive or negative impacts of agriculture on nutrition must act through these channels. Descriptively, we offer a health production function for nutritional outcomes:

$$H_{i}=H(f_{i}\text{,}n_{i}\text{,}s_{i}\text{,}X_{i})\text{,}$$

which over time accumulate as:

$$H_{\mathrm{it}}=H(f_{\mathrm{it}}\text{,}n_{\mathrm{it}}\text{,}s_{\mathrm{it}}\text{,}X_{i}\text{,}H_{\mathrm{it}-1})$$

where time *t-*indexed food consumption $f_{\mathrm{it}}$, care/nurturing $n_{\mathrm{it}}$, and sanitary environment $s_{\mathrm{it}}$ as well as a vector of individual or household characteristics $X_{i}$ and previous nutritional health outcomes $H_{\mathrm{it}-1}$. Lack of any factor, such as food, care, sanitation, may be sufficient to induce under-nutrition, and the provision of each is expected to complement the others in producing health (while competing through the budget constraint), so we would expect the true production function will contain interactions of these terms, likely with non-linearities and minimal subsistence terms.

Connecting the dots conceptually from income to nutrition, households may value health directly or may value consuming inputs that contribute to health (food, care, sanitation) as well as other consumption $c_{\mathrm{it}}$ and leisure $l_{\mathrm{it}}$, according to household characteristics $X_{\mathrm{it}}$:

$$U_{\mathrm{it}}=U(f_{\mathrm{it}}\text{,}n_{\mathrm{it}}\text{,}s_{\mathrm{it}}\text{,}c_{\mathrm{it}}\text{,}l_{\mathrm{it}}\text{,}X_{\mathrm{it}})\text{.}$$

The household wants to maximize utility subject to a budget constraint such as

$$p_{f}f_{\mathrm{it}}+p_{s}s_{\mathrm{it}}+p_{c}c_{\mathrm{it}}-w(n_{\mathrm{it}}+l_{\mathrm{it}})⩽I_{\mathrm{it}}$$

where $p_{f}\text{,}p_{s}\text{,}p_{c}\text{,}w$ are the prices of food, sanitation, other consumption, and the wage rate; and income $I_{\mathrm{it}}$ comprises farm profits, non-agricultural enterprise profits, and the value of household labor and land endowments.^5^

Under basic household models, income only affects these nutrition-inducing consumption choices by setting the budget constraint, with no other characteristic of income having influence. By relaxing the budget constraint, increases in income from any source may lead to greater food consumption; nutritional gains may be further facilitated by higher marginal consumption of food among the poor (Engel’s Law) especially in terms of consumption of calories and essential micronutrients (Skoufias et al., 2012, Strauss and Thomas, 1995, Subramanian and Deaton, 1996). At the same time, income gains enable greater consumption of complementary health inputs such as sanitation improvements and healthcare services, and the income elasticity of health and sanitation expenditures can remain quite high throughout the income distribution (Von Braun, de Hean, & Blanken, 1991). Income can be used for childcare services or otherwise improve the quality of care given as well. For example, higher expenditure on education allocated to girls as a result of increased income eventually translates into higher maternal education, shown to improve child nutritional outcomes (Behrman and Wolfe, 1984, Umapathi, 2008, Webb and Block, 2004), though this can take years or decades to materialize.

Empirical studies using pooled cross-sectional data provide evidence that nutritional outcomes do improve alongside long-run, aggregate economic growth (Cole, 2003, Haddad and Smith, 2002, Headey, 2013^6^; Webb & Block, 2010). Yet this relationship is not guaranteed, depending on duration and distribution of growth. Under the permanent income hypothesis and consumption smoothing, short-term income fluctuations may be less likely to induce consumption of food or sanitation when compared to longer term gains (Hall & Mishkin, 1982). Clearly, a household must be able to participate when there is aggregate growth in order to benefit from it. Looking at “nutritional episodes” with an average duration of 4.7 years, Heltberg (2009) looks at income growth across countries but finds less improvement in child stunting rates compared to longer term studies, with less nutrition improvement in more unequal societies. Relatedly, Webb and Block (2010), with data largely drawn from Sub-Saharan Africa, find that growth from structural transformation fails to support nutrition for the rural poor in the short run but point to agriculture effectively lowering stunting by reaching the rural poor. Headey (2013) finds that once India is excluded in cross-country regressions, agricultural growth corresponds to a stronger reduction in stunting than non-agricultural growth in the medium term. Again, even if a household is able to participate in income growth, conversion to nutrition through the mechanisms of food, care, and sanitation may take time.

### (a) Agriculture for income

Is agricultural growth the most effective way forward to support nutrition? These income results for nutrition above need not be specific to agricultural income. Yet since many of the world’s rural poor are dependent on agriculture as their main livelihood, growth in agriculture has the potential to be relatively more effective in reducing income poverty (Chen and Ravallion, 2007, de Janvry and Sadoulet, 2010), a strong determinant of under-nutrition. At the macro level, Ligon and Sadoulet (2007) find that agricultural income growth exerts a particularly beneficial effect on expenditures among the poorest and that non-agricultural growth boosts expenditures in a more modest fashion among these households. Also using cross-country studies, Heltberg (2009) suggests the explanation that non-agriculture growth associated with structural transformation tends to be geographically exclusive of the rural poor, and Loayza and Raddatz (2010) provide evidence that the gains arise through agriculture providing labor-intensive income opportunities to the unskilled. Christiaensen, Demery, and Kuhl (2011) find that the benefits from agricultural growth are more concentrated among the extreme poor (less than $1 a day) than among the better-off poor. Extending from poverty outcomes to nutrition outcomes, if agriculture is more accessible to the poor, then agricultural income could have more potential to improve nutrition-supporting consumption.

*Agricultural sub-sectors: commercialization vs. own consumption, and crop choice*. Within agriculture, too, the type of agricultural growth may have important implications for pass-through to nutrition. Agricultural commercialization is often favored for its ability to facilitate specialization, technological growth, and higher expected returns, thus allowing households to convert in-kind income to cash income, which can in turn be used to purchase greater food security and other health-supporting goods and services (Kennedy, 1994, Pingali, 1997, Pingali and Rosegrant, 1995, Romer, 1993, Romer, 1994, Timmer, 1997, Von Braun, 1995).^7^ Yet findings from various studies suggest that increased income through commercialization have not always yielded nutritional improvements and sometimes have been associated with nutritional declines among farming households (DeWalt, 1993, Dewey, 1981, Fleuret and Fleuret, 1980, Kennedy et al., 1992, Von Braun and Kennedy, 1986, Von Braun and Kennedy, 1994). Theoretical mechanisms for this possibility reflect that cash income can facilitate substitution toward non-food consumption or toward consumption of less nutritious foods through changing preferences or shifting of resources and/or control among household members with different expenditure preferences (Bouis and Haddad, 1990, Von Braun, 1995, Von Braun et al., 1991). Other studies offer an alternative explanation in which labor inputs necessary for commercialization may in some cases detract from health-supporting efforts in the home (e.g., breastfeeding or other childcare) (Abbi et al., 1991, Kennedy and Cogill, 1987, Popkin, 1980) or increase exposure to hazardous chemical inputs or zoonotic disease (Mullins, Wahome, Tsangari, & Maarse, 1996). Access to commercialization may also offer household investment opportunities that increase the opportunity costs of current consumption, potentially suppressing food expenditures in the short run.

By contrast, agricultural production for own consumption (subsistence agriculture) has been viewed traditionally as a last-resort, low-productivity option for those who face high transaction costs and missing markets or who are highly risk averse (Timmer, 1997). Yet there is a growing momentum for promoting own production as a direct support of food security, dietary diversity, and nutrient-dense consumption. An implicit assumption in these interventions is that food production income will be more likely to “stick” as food consumption relative to other kinds of income. For example, Von Braun *et al.* (1991) found that even after controlling for total income level, households with higher ratios of subsistence food production as a proportion of total income show higher food consumption. Designed with this stylized fact in mind, interventions that encourage dietary diversity and protein or micronutrient consumption through home-production channels—home gardens, biofortified varieties, and animal-sourced foods—do appear to successfully effect improvements in relevant biomarkers in some cases, with the caveats mentioned above (Masset *et al.*, 2011).

The “stickiness” of gains in own food production may bear out in part through price effects and risk aversion.^8^ The Food Price Crisis of 2007–08 has served as a reminder that the production of food crops can help insure vulnerable groups’ consumption against food price risk, since rising food prices also raise the income value of the crop at the same time (de Janvry and Sadoulet, 1995, Headey, 2013). Especially in rural areas, where households face shallow markets, seasons of high and geographically correlated production will lower relative food prices, inducing substitution toward food consumption. Price risk aversion and transaction costs can further increase consumption of own production by driving a wedge between the effective sale and purchase prices, again making consumption of own food relatively more attractive (de Janvry et al., 1991, Jensen, 2010, Key et al., 2000, Svensson and Yanagizawa, 2009). Completely missing markets for the purchase of nutritious foods represent the extreme case of transaction costs, in which the only means of acquiring necessary micronutrients and achieving dietary diversity is own production.

In a more mechanical sense similar to the general argument for income, improved productivity in food cropping for own consumption may be differentially good at boosting food consumption and then nutritional outcomes because it is often the very poor and women who engage in subsistence agriculture and who may be most likely to convert gains into increased food intake.

Aside from the distinction between commercialization and own production, the nutritional qualities of the particular crop (or animal) associated with income growth may also be relevant. At the macro level, Headey (2013) goes further than previous cross-country analyses to show that the nutritional gains are strongest where agricultural growth manifests as increased food production and in countries whose food production was low initially.^9^ And to explain part of India’s failure to convert economic growth to nutrition, pooled cross-sectional studies point to non-food agricultural production and price effects that shift consumption from more protein-rich pulses toward cheaper and less nutrient-rich grain (Deaton and Drèze, 2009, Headey et al., 2012). Given the level of geographic aggregation, however, none of these studies are able to offer insight into whether for the individual or the households, it is important to produce for one’s own consumption, or whether in the presence of sufficiently deep markets for nutritious foods, households may be better of maximizing the income value rather than the nutritional value of their agricultural portfolios. Clearly, the nutritional benefit derived from consumption from own crop production will depend on the nutritional quality of crops being produced, and the benefit from other sources of income must depend on the nutritional value of food being purchased.

## 3 Data

To explore the links between income and child nutrition, we make use of three rounds of the nationally representative Uganda National Panel Survey (UNPS) collected in 2009–10, 2010–11, and 2011–12. The UNPS is implemented by the Uganda Bureau of Statistics with financial and technical support from the World Bank Living Standard Measurement Study—Integrated Surveys on Agriculture (LSMS-ISA) program.^10^ In each round, the survey collects anthropometric measures (height and weight) for children under five years of age and detailed information on household consumption, income, and agricultural activities. Having individual-level anthropometric measures and household-level income, including detailed information about agricultural production and consumption from own production, offers the opportunity to explore potential pass-through (or not) from income to nutrition via consumption.^11^ Specific to the UNPS, the panel nature (at the household and individual levels) and the short period between the survey rounds allow us to conduct within-household analyses over time, and to control in our estimations for unobserved time-invariant child attributes that may be missed in cross-sectional or pooled cross-sectional investigations. In fact, it is this feature that provides the basis for the present study.

Given the different income and consumption patterns between urban and rural populations, the specific focus on agricultural income, and the relatively smaller sample size for the urban population, we focus on the rural subsample of households in each round and on children that appear in at least two of the three survey rounds. The latter restriction allows for the inclusion of child-specific fixed effects in our estimations, and leads to 748, 924, and 653 child observations in 2009–10, 2010–11, and 2011–12, respectively, to be part of the analysis sample. Of these, 326 were observed in all three panel years. 671 children were observed in two years: 341 for the first two years, 251 for the last two years, and 78 for the first and last year.

Table 1a provides summary statistics for the sample across the three panel waves. Within this sample, the median household has approximately seven members, including four children under the age of 15 and two children less than five years of age. 53% of the children in the sample are male. As is typically found in Uganda, there are much higher levels of stunting (35–38 percent across three years) than underweight (11–16%).

**Table 1a:** Summary Statistics for UNPS 2009–10–2011/12

Demographics, Assets, and Child Characteristics Subset: rural children with at least two observations in the three waves									
	2009–10 (748 obs)			2010–11 (924 obs)			2011–12 (653 obs)		
Variable	mean	sd	median	mean	sd	median	mean	sd	median
REGION									
Central w/o Kampala	0.235	0.424	0	0.256	0.437	0	0.270	0.444	0
Eastern	0.320	0.467	0	0.318	0.466	0	0.291	0.455	0
Northern	0.287	0.453	0	0.302	0.459	0	0.309	0.463	0
Western	0.158	0.365	0	0.123	0.329	0	0.130	0.337	0

DEMOGRAPHICS									
Household size	7.13	2.78	7	7.04	2.56	7	7.11	2.56	7
Number of children < 15 yrs	4.33	1.93	4	4.37	1.97	4	4.36	1.96	4
Number of children < 5 yrs	1.95	0.809	2	1.94	0.835	2	1.94	0.886	2
Number of adults 15+	2.81	1.43	2	2.67	1.21	2	2.75	1.24	2
Number of males in the household	1.26	0.944	1	1.19	0.804	1	1.26	0.821	1
% Female-headed household	0.168	0.375	0	0.180	0.384	0	0.176	0.381	0
Dependency ratio	1.83	0.992	1.59	1.95	1.12	1.67	1.90	1.12	1.67
Head's years of school	5.61	4.32	6	6.28	4.63	6	5.75	4.32	6
Spouse's years of education	3.88	3.77	4	4.51	4.24	4	4.26	3.93	4
Average years of educ. for members > 21	4.54	3.15	4	5.01	3.45	4.4	4.60	3.16	4
Highest years of education in household	7.17	4.26	7	7.99	4.64	7	7.38	4.2	7

ASSETS									
Has improved roof	0.523	0.500	1	0.527	0.500	1	0.545	0.498	1
Has improved walls	0.634	0.482	1	0.647	0.480	1	0.688	0.464	1
Has improved floor	0.148	0.356	0	0.162	0.369	0	0.164	0.37	0
Treats water	0.357	0.479	0	0.339	0.474	0	0.315	0.465	0
Has improved water source	0.686	0.464	1	0.697	0.460	1	0.741	0.438	1
Has improved toilet facility	0.893	0.309	1	0.878	0.328	1	0.888	0.315	1
Has hand washing station	0.098	0.297	0	0.042	0.201	0	0.075	0.264	0

CHILD									
Age of child (in months)	25.2	11.5	25	33.1	14.3	33	40.5	11.1	41
Gender: 1 = male	0.528	0.5	1	0.531	0.499	1	0.531	0.499	1
Height, cm	81.4	9.33	81.4	86.8	10.7	87	91.4	8.19	91
Weight, kg	10.8	2.56	10.6	12.3	2.92	12.4	13.5	2.43	13.4
Height-for-Age score (HAZ)	−1.55	1.48	−1.59	−1.58	1.40	−1.56	−1.71	1.25	−1.69
Weight-for-Age score (WAZ)	−0.91	1.17	−0.855	−0.79	1.10	−0.775	−0.82	1.02	−0.81
Weight-for-Height score (WHZ)	−0.08	1.18	0.015	0.138	1.13	0.14	0.216	1.09	0.21
% Stunted (HAZ < −2)	0.368	0.482	0	0.354	0.478	0	0.380	0.486	0
% Wasted (WHZ < −2)	0.159	0.366	0	0.116	0.32	0	0.104	0.306	0
% Underweight (WAZ < −2)	0.059	0.236	0	0.031	0.175	0	0.020	0.140	0

*Notes:* Sample includes only rural households with children whose birthdate was consistent across panel waves. *z*-Scores calculated from date of birth rather than reported age in months when not in agreement.

Table 1b presents income and consumption statistics for the sample, deflated to the first survey round in 2009–10 and converted to US dollars. While rural households derive their income from multiple sources, nearly all households participate in crop farming in each round, and the vast majority also engages in livestock activities. Approximately half report non-agricultural self-employment, with average self-employment (for the whole population) approximately equal to average crop income. Agricultural and non-agricultural wage employment each count one-quarter of the households, with greater income coming from non-agricultural wages.

**Table 1b:** Summary Statistics for UNPS 2009/10–2011/12

Income and Consumption Subset: rural children with at least two observations in the three waves									
	2009–10 (748 obs)			2010–11 (924 obs)			2011–12 (653 obs)		
Variable	mean	sd	median	mean	sd	median	mean	sd	median
INCOME									
Net total income (1)	892	1198	578	968	1085	623	1105	1416	703
Net total income (2)	1084	1215	775	1075	1100	764	1230	1420	893
Gross total income (1)	1399	1679	870	1459	1578	954	1548	1891	988
Gross total income (2)	1589	1705	1103	1569	1587	1076	1673	1884	1153

PARTICIPATION									
% Agriculture wage employment > 0	0.286	0.452	0	0.237	0.425	0	0.193	0.395	0
% Non-ag wage employment > 0	0.242	0.429	0	0.215	0.411	0	0.225	0.418	0
% Crop production (1) > 0	0.939	0.240	1	0.926	0.261	1	0.928	0.259	1
% Crop production (2) > 0	0.949	0.220	1	0.944	0.231	1	0.939	0.240	1
% Livestock production > 0	0.762	0.426	1	0.728	0.445	1	0.689	0.463	1
% Non-ag self employment > 0	0.537	0.499	1	0.522	0.500	1	0.489	0.500	0
% Transfers > 0	0	0	0	0	0	0	0.006	0.078	0

SECTOR INCOME									
Self-employ, net	192	585	3.55	264	641	1.84	292	762	0
Self-employ, gross	494	1242	22.8	559	1215	26.2	604	1399	0
Crop income (1), net	282	416	171	279	349	178	373	506	234
Crop income (2), net	358	456	226	357	381	252	441	546	275
Crop income (1), gross	474	466	386	386	389	295	499	524	395
Crop income (2), gross	548	502	442	467	416	361	565	559	447
Livestock income, net	115	237	53.2	127	252	63	91.7	188	29.8
Livestock income, gross	242	281	149	239	290	146	150	210	74.5
Wages	294	886	0	287	753	0	332	1056	0
Ag wages	84.6	263		61.3	176		71.3	256	0
Non-ag wages	210	820		226	746		260	1034	0

CROP SHARES									
% Gross income from crops	0.355	0.285	0.289	0.360	0.295	0.292	0.401	0.313	0.345
% Gross crop income, low-protein crop	0.306	0.302	0.237	0.333	0.294	0.290	0.340	0.309	0.310
% Gross income, low-protein crop	0.121	0.168	0.051	0.136	0.177	0.069	0.160	0.193	0.087
% Gross crop income, other food crop	0.575	0.342	0.604	0.529	0.336	0.527	0.531	0.340	0.539
% Gross income, other food crop	0.210	0.217	0.139	0.195	0.206	0.131	0.215	0.218	0.137
% Gross crop income, nonfood crop	0.057	0.141	0	0.067	0.146	0	0.057	0.136	0
% Gross income, nonfood crop	0.024	0.068	0	0.028	0.070	0	0.026	0.068	0

CONSUMPTION									
Total annual food consumption	694	509	579	835	619	685	804	640	639
Total annual crop food consumption	246	196	196	261	221	194	256	236	201
Total annual low-protein food consumption	27.6	58.5	0	25.7	61.8	0	36.1	74.5	0
Total annual livestock/byproduct consumption	97.5	118	51	131	145	90.3	135	149	96.8
Total annual food consumption, purchases	333	251	272	400	307	321	380	310	308
Crop income, own consumption (1)	170	176	112	180	175	131	229	233	153
Crop income, own consumption (2)	360	266	313	294	246	239	360	280	329
Low-protein crop income, own consumption (1)	90.6	127	43	99.4	125	55.8	131	163	71.7
Low-protein crop income, own consumption (2)	126	134	89.2	119	160	71.8	126	147	67.8
Livestock income, own consumption (2)	43.1	93.2	0	52	112	0	44.5	102	0

*Notes:* Sample includes only rural households with children whose birthdate was consistent across panel waves. Total income and crop income (1) with value of consumption from own production calculated from agricultural module. Total income and crop income (2) calculated from consumption module. All income and consumption values are deflated to 2009–10 and converted to USD using March 1, 2010. “Low-protein crops” are cassava and plantain, two staple food crops in Uganda.

We report initially two alternative crop income calculations that include the estimated value of household crop production that was consumed at home either from (1) the agriculture questionnaire or (2) the food consumption section of the household questionnaire.^12^ The second methodology generates lower total and crop income in the second wave compared to the other rounds: this reflects local and international food price fluctuations that occurred during the span of the survey periods more than changes in production. The second methodology also relies on consumption data from a single week, while the first methodology uses reported consumption of own produce for the entire year.^13^ To minimize the impact of seasonality in food consumption reporting on the calculation of income measures, we elect to focus on the crop and total income variables derived using the first methodology for the remainder of the paper.^14^

More than one-third of gross income comes from crops, with approximately one-third of crop income coming from two “low-protein” crops, namely cassava and plantain varieties. We do not classify other crops according to nutritional status but focus on cassava and plantain both as the top two starchy staples consumed in Uganda as well as two major crops that are particularly low in protein as well as many other important nutrients (FAO, 1990), in a context where diets are recognized to be largely deficient in protein and vitamin A and zinc, among other micronutrients (FANTA/USAID, 2010). These two crops account for 12–16% of total gross income. Non-food crops, which include coffee, comprise only a small fraction of the gross income portfolio. The decrease in livestock income in the third year is tied to the decline in the sales, births, and production of byproducts, which may partially be underlined by the outbreak of food and mouth disease during the reporting period (FEWS NET, 2011).^15^

To examine the implications of restricting the sample for fixed effects to include only children who appear in at least two of the three survey rounds,^16^ the in-sample and out-of-sample means were compared for the children in the first survey round (2009–10) for each of the variables reported in Table 1a, Table 1b. Table 1c reports only the outcomes for which the differences were statistically significant at the 0.10 level or lower. The included sample is more heavily representative of the Eastern Region, and less representative of the Western Region. The spouse of the head of household tends to have completed a half a year more of schooling. The included sample is slightly more representative of boys, at 53% versus 49. Unsurprisingly, the included sample is an average of 13 months younger in the first survey round. In fact, approximately 40% of the sample was over the age of 48 months during the first round; most of these would be too old to be measured in any following round and thus drop from the sample. Due the age difference, weight and height are also lower among the included sample; however the respective *z*-scores are not statistically different. Many of the remaining differences suggest that more heavily agricultural households were more likely to be resurveyed in future rounds, potentially related to greater permanence of residence and lower attrition.^17^

**Table 1c:** Comparative statistics

In Sample vs Out of Sample, 2009/2010 Survey Wave Subset: Rural children								
	Means		Difference			Means		Difference
	In Sample	Out of Sample				In Sample	Out of Sample	
REGION				ASSETS				
Eastern	0.32	0.268	0.052^**^	Has improved roof		0.522	0.598	−0.075^***^
	(0.02)	(0.02)	(0.02)			(0.02)	(0.02)	(0.02)
Western	0.157	0.256	−0.098^***^	Has improved walls		0.63	0.55	0.07^***^
	(0.01)	(0.02)	(0.02)			(0.02)	(0.02)	(0.02)
DEMOGRAPHICS				PARTICIPATION				
Spouse's years of education	3.89	3.41	0.47^**^	% Crop production (1) > 0		0.94	0.88	0.06^***^
	(0.14)	(0.13)	(0.19)			(0.01)	(0.01)	(0.01)
CHILD				% Crop production (2) > 0		0.95	0.90	0.046^***^
Age of child (in months)	25.1	38.0	−12.9^***^			(0.01)	(0.01)	(0.01)
	(0.42)	(0.58)	(0.71)	% Livestock production > 0		0.76	0.72	0.04^*^
Gender: 1 = male	0.53	0.49	0.041^*^			(0.02)	(0.02)	(0.02)
	(0.02)	(0.02)	(0.03)	SECTOR INCOME				
Weight, kg	10.8	13.0	−2.13^***^	Livestock income, net		116.0	96.0	20.0^*^
	(0.09)	(0.11)	(0.14)			(8.69)	(8.17)	(12.0)
Height, cm	81.3	89.5	−8.2^***^	Ag wages		84.0	60.6	23.4^**^
	(0.35)	(0.45)	(0.59)			(9.5)	(6.6)	(11.3)
				% Gross crop income,		0.57	0.52	0.05^***^
				other food crop		(0.01)	(0.01)	(0.02)

*Notes:* Only significant differences are presented, from comparisons of all variables presented in Table 1a, Table 1b. Std errors in parentheses. In Sample includes children in at least two rounds, with matching or reconcilable birthdates and income data. Sample size: In Sample, 756 obs; Out of Sample, 895 obs.

**Figure 1:**
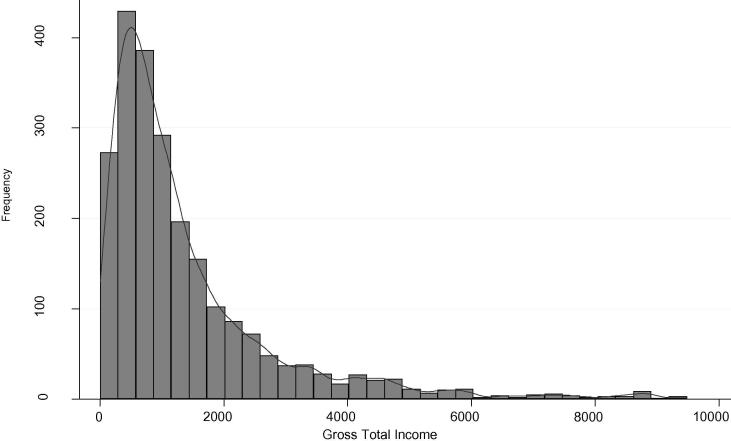
Distribution of income levels (USD).

**Figure 2:**
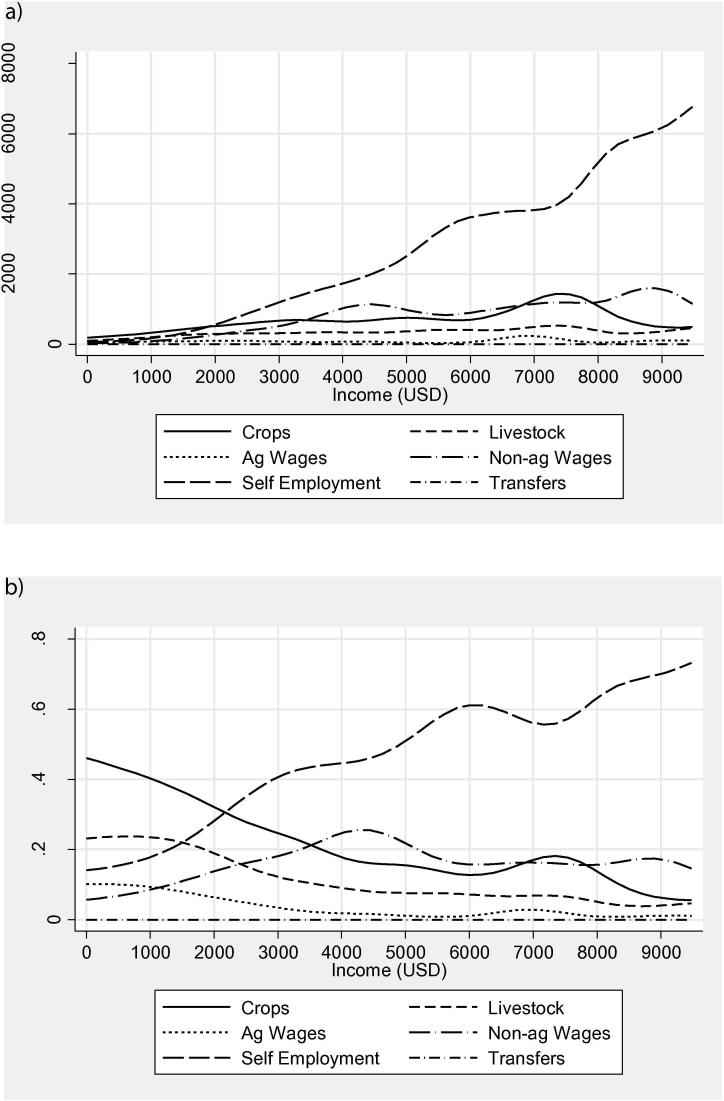
(a) Income subtypes by total income (USD). (b) Income shares by income level (USD).

**Figure 3:**
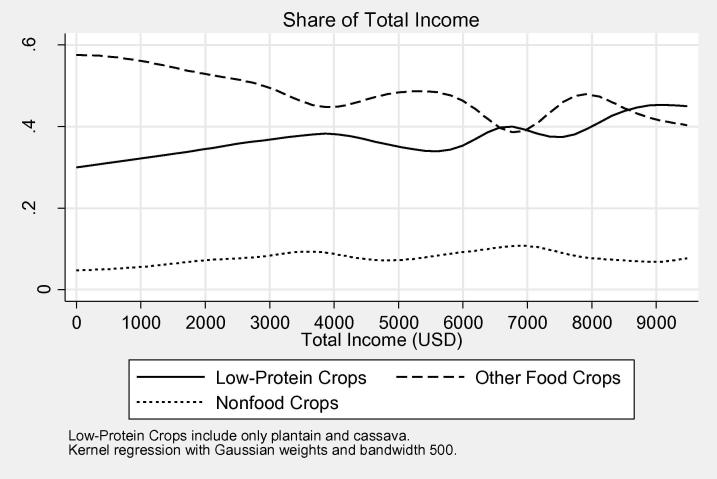
Income share from crop subtypes by total income.

Figure 1 gives the distribution of gross total income for all three year combined.^18^ Figure 2a and b give a bit more insight into the distribution of income by source. Though income from any particular source does not decrease in levels, as overall income increases, there is a clear pattern of agricultural income (crops, livestock, and agricultural wages) falling as a share of total income, strongly in favor of self-employment income. Except for the top percentage of earners, nonagricultural wage also increases its contribution as total income increases. Breaking down crop income into three types, low-protein (cassava and plantain varieties), non-food (cotton, tobacco, coffee), and other food crops, Figure 3 shows a trend that low-protein crops and non-food crops constitute an increasing percentage of crop income among higher crop income earners, though the sample of farmers who grow nonfood crops at any income level is small.

### (a) Nutritional outcomes

**Figure 4:**
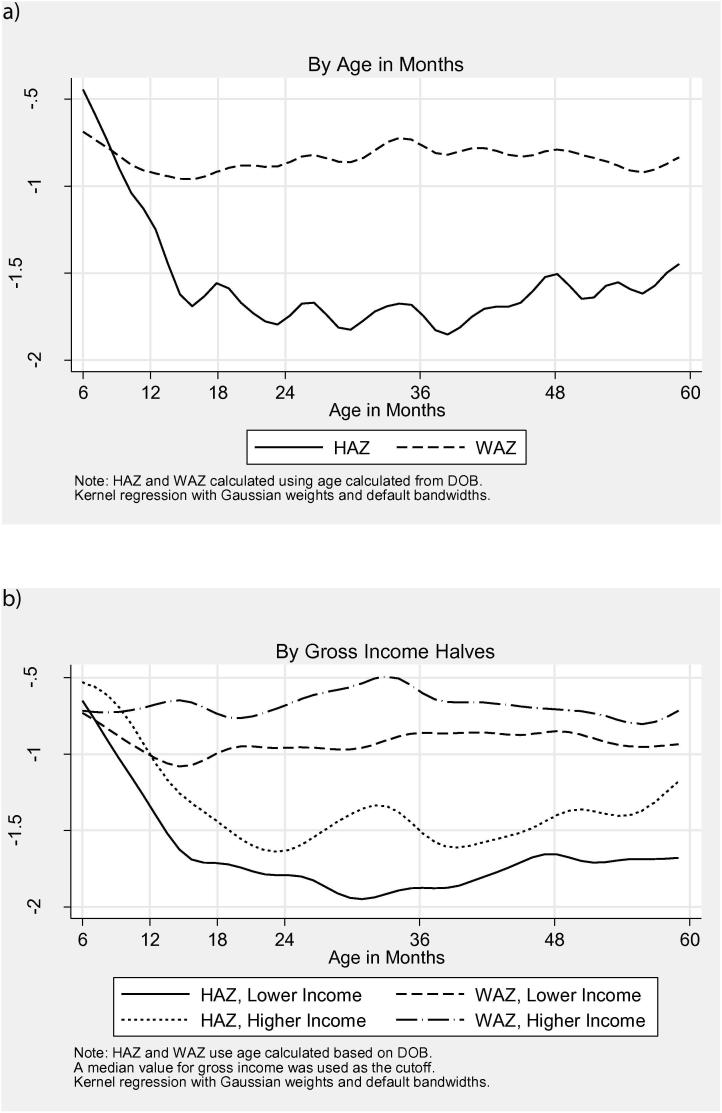
(a) Height-for-age and weight-for-age *z*-scores. (b) Height-for-age and weight-for-age *z*-scores.

For our outcomes of interest, we use children’s anthropometric measurements to reflect their nutritional status. Because we use differences in income and anthropometry over one-year periods, we focus specifically on height, which is considered to be the best measure of long-term growth. According to the World Health Organization guidelines, we use height-for-age (HAZ) *z*-scores to normalize height measures by age in order to allow for useful comparisons across children of various ages.^19^^,^^20^ Figure 4a presents kernel regressions of HAZ and WAZ (weight for age) on age in months for children between the ages of 6 and 59 months in the rural UNPS sample. Nutritional challenges are readily apparent: average height for age plunges quite steeply during the first 18 months and remains low. While both WAZ and HAZ are below international norms, it is striking that HAZ in particular is more than 1.5 standard deviations below international norms throughout childhood suggesting a diet that is less energy deficient (captured by WAZ) and more nutrient deficient. Given the strong nonlinearities over time, in many specifications we will opt to include flexible controls for age (Cummins, 2015).

To begin looking at the static relationship between income and nutritional status, Figure4b presents kernel regressions of HAZ and WAZ on age in months split by median income.^21^ The top two kernel density plots show weight for age *z*-scores, which stay relatively flat across ages. The higher plot marks children whose households are above the median income for the sample; the poorer half the sample tracks the same relatively flat trajectory but at a lower score. For height for age, both groups decline during the first two years, but the higher income half declines somewhat less dramatically. In the absence of omitted variables, a first glance would lead us to expect a strong correlation between income and nutritional status.

**Figure 5:**
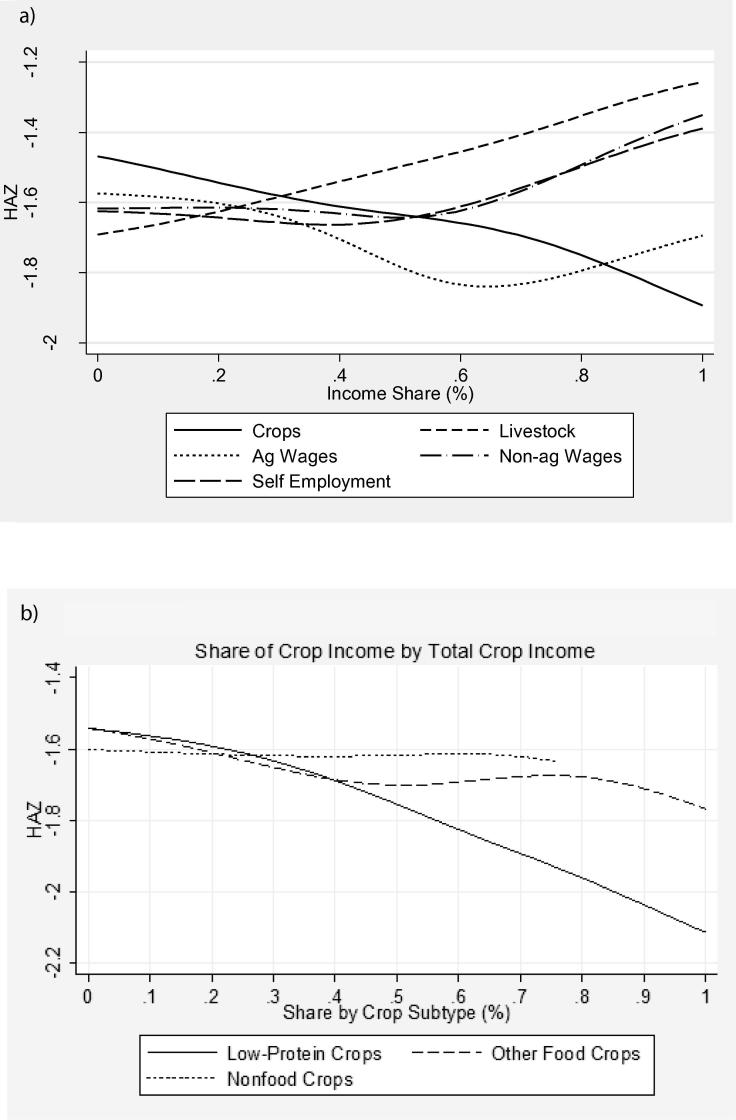
(a) HAZ by income shares. (b) HAZ and income share from crop subtypes.

Figure5a show**s** HAZ by income shares by source. Households move toward the right on any curve if they specialize in that sector. The highest *z*-scores are among those most specialized in livestock, with non-agricultural self-employment and non-agricultural wage also looking favorable relative to crops and agricultural wages. These do not speak to changes in income nor total levels of income but might inform initial priors. Similarly, Figure5b shows *z*-scores by shares of crops by subtype as a proportion of gross total income. The lowest average *z*-scores are among those with the highest shares of income from low-protein crops. These figures, however, only describe anthropometric trends based on a static income profile and do not show that increases in low-protein crop income would lower *z*-scores.

While our dataset is very rich in a number of ways, there are some missing elements that might allow for deeper analysis than what we are able to conduct presently. First, while there is a consumption module for the household, there is no individual-level consumption data. Given that young children are frequently fed a different mix of foods from the rest of the household, it is difficult to infer children’s consumption from the household data. For this reason, data collected specifically for research on children’s nutrition will collect information on children’s diet specifically as well as on the frequency of feeding. There is also no module on time use or other information that might be able to link income or labor activities more carefully to childcare tasks, another theoretical mechanism through which certain types of income could lead to differential outcomes for children.

## 4 Empirical strategy

Using the subset of households in the UNPS with children under the age of five described above, we test a set of hypotheses suggested by the theory laid out in the first sections, taking advantage of the child-level panel data. We show our most basic results on income without and then with child fixed effects, and then proceed with the preferred specification with child fixed effects.

We first look for evidence on the central question of whether short-term changes in income correspond to observable changes in nutritional outcomes. As a benchmark, we begin with the estimation most available in the literature, treating our panel dataset as a set of repeated cross sections. Thus, we estimate:

(1) $$H_{\mathrm{it}}=\beta_{Y}\log Y_{\mathrm{it}}+{∊}_{\mathrm{it}}\text{,}$$

where $H_{\mathrm{it}}$ represents the health measure (height for age (HAZ) *z*-score, height in centimeters, weight for age (WAZ) *z*-score, and weight in kilograms); $Y_{\mathrm{it}}$ is income, which can be specified various ways; and ${∊}_{i}$ is the error term. We use the log of total gross income for interpretation in percentages and to accommodate diminishing marginal returns, after finding qualitatively similar results with a combination of level and square root of gross income. However, there is a possibility of omitted variable bias from observable and unobservable characteristics that may influence both income and the anthropometric measures of children (parental education as one likely candidate). A vector of additional covariates $Z_{i}$ may be added to capture such observable characteristics along with a survey round fixed effect $\eta_{\text{t}}$ and seasonal (month) fixed effect $s_{t}$ to absorb unobservable characteristics that are common to the sample in a particular survey round or a certain month of the year:

(2) $$H_{\mathrm{it}}=\beta_{Y}\log Y_{\mathrm{it}}+Z_{i}\gamma +\eta_{t}+s_{t}+{∊}_{\mathrm{it}.}$$

Still, there are likely other unobservable (or simply unobserved) attributes that may bias the estimation results. One example might be tall parents whose height and strength increase wage earnings but also genetically predispose a child to attain greater height or weight than average. Another example could be related to parental intelligence, which can be used for earnings and for providing better care for children. In the absence of a set of convincing instruments for income variables, including a child-specific fixed effect in the specification allows us to control for time-invariant child, household, and community characteristics that might otherwise jointly determine income and child nutrition outcomes.^22^

Thus, to test whether we observe changes in short-term changes in children’s nutrition corresponding to short-term total income, we estimate:

(3) $$H_{\mathrm{it}}=\beta_{Y}\log Y_{\mathrm{it}}+Z_{i}\gamma +\eta_{t}+s_{t}+\nu_{i}+{∊}_{\mathrm{it}}\text{,}$$

where $H_{\mathrm{it}}$ represents the health measure (height for age (HAZ) *z*-score, height in centimeters, weight for age (WAZ) *z*-score, and weight in kilograms), $Y_{\mathrm{it}}$ is the total gross income, $\eta_{t}$ is a survey round fixed effect, and $\nu_{\text{i}}$ is a child fixed effect, and ${∊}_{i}$ is the error term. Here, the vector of covariates $Z_{i}$ is limited to include child age, with age fixed effects that allow a flexible specification by month (Cummins, 2015), female headship, number of children, and total household size. Even with child fixed effects, however, there remains a possibility of time-variant unobservable factors that may bias these estimates. Therefore, we see our estimation as a useful and informative diagnostic exercise but caution overly strong confidence in causal interpretations.

### (a) Income by sector

The next step is to test whether changes in height are related to the sector of income. We conduct this analysis by looking for differential impacts of income from sectors *vis-à-vis* total income. We start by adding sector share of total income for the each of the four major income sectors individually: crops, livestock, nonagricultural self-employment:

(4) $$H_{\mathrm{ti}}=\beta_{s}(Y_{\mathrm{it}}^{s}/Y_{\mathrm{it}})+\beta_{Y}\text{log}Y_{\mathrm{it}}+\eta_{t}+\nu_{i}+{∊}_{i}$$

where $\left(Y_{\mathrm{it}}^{s}/Y_{\mathrm{it}}\right)$ is the share of gross income coming from sector *s* and $Y_{\mathrm{it}}$ is the total gross income.^23^ The term for total income is included to avoid omitted variable bias that would result if changes different income shares are correlated to changes in total income. In this specification, if consumption and nutrition are only related to income through the budget constraint, we would expect to see no statistically significant coefficients for the sector indicators. This is the cleanest and most straightforward to interpret, as share of each sector compared to the *sum of all other sectors*.

Further, the preferred specification that simultaneously assesses the relative effects of different sectoral income shares and that avoids picking up any correlations between the different sources includes sector share for three of the four major income sectors at the same time: crops, livestock, nonagricultural self-employment. In this case, using wage labor as the omitted (fourth) category, we estimate:

(5) $$H_{\mathrm{it}}=\Sigma_{\text{s}}\beta_{s}(Y_{\mathrm{it}}^{s}/Y_{\mathrm{it}})+\beta_{Y}\log Y_{\mathrm{it}}+\eta_{t}+\nu_{i}+{∊}_{i}\text{.}$$

This specification will show different coefficients if changes in income are especially correlated between certain sectors more than others.^24^

*Income by crop type.* We test in the Ugandan context the idea commonly seen in the literature that type of crop income may influence nutrition. To do so, we break crops into two broad categories based on nutrient availability: (1) low-protein food crops and (2) other crops.^25^ We adopt the same shares approach used for sector income, looking at the share of each crop type *a* in total income:

(6) $$H_{\mathrm{it}}=\beta_{a}(Y_{\mathrm{it}}^{a}/Y_{\mathrm{it}})+\beta_{Y}\log Y_{\mathrm{it}}+\eta_{t}+\nu_{i}+{∊}_{i}$$

Again, we add controls for total income, and consider specifications that add total crop share. It is important to note that the two crops we categorize as low-protein are banana/plantain and cassava—the top two staple crops in Uganda (and grown by more than 70% of our sample) with lower protein availability than other staples such as cereals and sweet potatoes. Uganda is recognized to maintain a low-protein diet. As such, income gains that come in the form of additional low-protein food might be less likely to benefit nutrition than other forms of income unless the produce is sold to fund other nutrition-supporting purchases.^26^

Finally, we attempt to address the question of whether agriculture may be impactful through production alone or more specifically through own consumption. We do so by comparing the above specifications on shares of crop production to specifications that include shares of own consumption of production, represented by

(7) $$H_{\mathrm{it}}=\beta_{c}(C_{\mathrm{it}}^{a}/Y_{\mathrm{it}})+\beta_{Y}\log Y_{\mathrm{it}}+\eta_{t}+\nu_{i}+{∊}_{i}$$

with $C_{\mathrm{it}}^{a}$ representing the value of consumption originating from own production.

## 5 Results

Table 2 shows the results for overall income for height-for-age (HAZ) and prevalence of stunting (HAZ < −2), respectively. The first two columns present HAZ *z*-scores as dependent variables, and the last two present stunting. Columns (1) and (3) show the standard pooled-cross sectional results with additional time-varying covariates and with standard errors clustered at the household level. These estimates represent the status quo for observational data where the same children cannot be followed over time. An income coefficient of 0.108 for HAZ is statistically significant but small, since we can interpret the coefficient to be the change in HAZ if household income doubles. For our sample, this only translates into approximately 0.08 standard deviations for a 100% gain in income. For the same change in income, we predict that the prevalence of stunting would fall by 3.8 percentage points, or approximately 10%. Coefficients for the additional covariates are not reported, for brevity, but these variables include time indicators for Round 2 and Round 3, child gender (only relevant for the pooled cross-sectional estimations), age-in-months fixed effects to accommodate the common non-linear fall of *z*-scores over time (as observed in Figure 4), interview month fixed effects to reduce seasonally based statistical noise in income reporting or child health, household size, number of children under 5 and under 15, household head years of education, and an identifier for and female headship.

**Table 2:** Height-for-age and stunting on total income and controls

Whole Sample (2325 observations in 757 clusters)				
	HAZ		% Stunting	
	(1)	(2)	(3)	(4)
Ln Gross Total Income	0.108^***^	0.0473	−0.0376^***^	0.00243
	(0.0389)	(0.0352)	(0.0131)	(0.0113)
**Child Fixed Effects**	NO	YES	NO	YES
***R*-Sq**	0.0926	0.229	0.0626	0.132
**Adj *R*-Sq**	0.0631	0.204	0.0322	0.104

Sub-sample: Under 24 Months (1069 observations in 496 clusters)				

	HAZ		% Stunted	
	(1)	(2)		

Ln Gross Total Income	0.119^**^	0.0908^*^	−0.0418^***^	−0.00129
	(0.0470)	(0.0541)	(0.0157)	(0.0163)
**Child Fixed Effects**	NO	YES	NO	YES
***R*-Sq**	0.130	0.334	0.0846	0.162
**Adj *R*-Sq**	0.0909	0.304	0.0430	0.123

Sub-sample: Below Median Income in the First Year (1574 observations in 577 clusters)				

	HAZ		% Stunted	
	(1)	(2)	(3)	(4)

Ln Gross Total Income	0.0880^*^	0.0374	−0.0228	0.00601
	(0.0491)	(0.0362)	(0.0178)	(0.0132)
**Child Fixed Effects**	NO	YES	NO	YES
***R*-Sq**	0.0930	0.310	0.0639	0.170
**Adj *R*-Sq**	0.0484	0.276	0.0179	0.129

*Notes:* Standard errors in parentheses, clustered at the household level; ^*^p < 0.1, ^**^p < 0.05, ^***^p < 0.01; All regressions include the following time-varying controls: indicators for the survey rounds, household size, number of children under 15 and under 5, identifier for female headed household, head's education, survey month fixed effects, and age-in-months fixed effects.

The preferred fixed effects specifications are presented in columns (2) and (4) of Table 2 with the same set of controls. In contrast to the pooled cross-sectional results, the coefficients on income in fixed effects estimations fall by more than half for HAZ and to nearly zero for stunting. Though the cross-sectional effects were fairly small, we can infer that unobservable characteristics drive some of the relationship between income and anthropometric outcomes that we are able to control for by comparing children to themselves over time. These results hold for subsamples below the sample median income in 2009–10 (i.e., the first survey round), but for the subsample below 24 months of age, the income coefficient holds fairly steady for HAZ. Again, this coefficient is quite small, but it may suggest that short-term income gains may have a small nutrition-supporting role for younger children, who are still in the most critical period of nutritional development (UNICEF, 1990, p. 21–23). Still, this effect is too small to suggest policy dependence on income growth to boost child growth measures. After this, all reported findings are based on regressions that control for child fixed effects and the aforementioned time-varying observables.

### (a) Income by sector

Table 3a presents the results for income shares by sector with HAZ as the dependent variable across the whole sample. We can interpret the coefficients as being the expected change in HAZ score if a household went from no crop income to 100% crop income. The crop income share exerts a negative and significant impact. In the more realistic change of 10–20% change in income share from year to year, the coefficient of −0.22 would translate to approximately 0.02–0.04 points of HAZ. On the other hand, the coefficient for self-employment is positive and highly significant, and slightly larger in magnitude than that of crop share, potentially reflecting self-employment enabling different consumption or care habits, and meriting further investigation in the future. Lastly, both livestock and wage shares have coefficients near zero.

**Table 3a:** HAZ on income shares (shares of gross sector income and child fixed effects)

Whole Sample (2325 observations in 757 clusters)				
	(1)	(2)	(3)	(4)
% Income, Crop Production	−0.215^*^			
	(0.118)			

% Income, Livestock		−0.0279		
		(0.128)		
% Income, Self-Employment			0.255^**^	
			(0.124)	

% Income, Wage Employment				−0.0133
				(0.128)

Ln Gross Total Income	0.0210	0.0463	0.0236	0.0481
	(0.0383)	(0.0358)	(0.0335)	(0.0389)

**Joint *P*-value**	0.0706	0.388	0.0906	0.371
***R*-Sq**	0.231	0.229	0.232	0.229
**Adj *R*-Sq**	0.206	0.204	0.207	0.204

*Notes:* Standard errors in parentheses, clustered at the household level; ^*^p < 0.1, ^**^p < 0.05, ^***^p < 0.01; Joint *P*-value is associated with the test of joint significance of the income shares and log gross total income; all regressions include the child fixed effects and the following time-varying controls: indicators for the survey rounds, household size, number of children under 15 and under 5, identifier for female headed household, head's education, survey month fixed effects, and age-in-months fixed effect.

### (b) Income by crop type

Table 3b presents the results for crop production and consumption of own crop production as shares of gross total income for the whole sample. We present total crop production and consumption and then the breakdowns by crop category. The coefficients can be interpreted as the predicted change in *z*-score from a change from no income coming from that source to 100% coming from that source.

**Table 3b:** HAZ—crop production and own consumption (shares of gross total income and child fixed effects)

Whole Sample (2325 observations in 757 clusters)								
	(1)	(2)	(3)	(4)	(5)	(6)	(7)	(8)
% Income, Crop Production	−0.215^*^				0.0899^*^	−0.0284	−0.0673	0.0348
	(0.118)				(0.180)	(0.142)	(0.141)	(0.186)

% Income, Consumption of Own Crop Production		−0.440^**^			−0.530^**^			−0.244
		(0.171)			(0.265)			(0.274)

% Income, Low-Protein Crop Production			−0.442^**^			−0.416^*^	0.159	0.156
			(0.189)			(0.232)	(0.314)	(0.310)

% Income, Consumption of Own Low-Protein Crop Production				−0.619^***^			−0.721^***^	−0.590^*^
				(0.225)			(0.343)	(0.335)

Ln Gross Total Income	0.0210	0.00187	0.0304	0.0214	0.00357	0.0279	0.0304	0.00771
	(0.0383)	(0.0415)	(0.0363)	(0.0371)	(0.0411)	(0.0383)	(0.0363)	(0.0418)

**Joint *P*-value**	0.0706	0.00943	0.0238	0.00765	0.0239	0.0562	0.0449	0.0570
***R*-Sq**	0.231	0.235	0.234	0.236	0.235	0.234	0.237	0.237
**Adj *R*-Sq**	0.206	0.209	0.209	0.211	0.209	0.208	0.211	0.211

*Notes:* Standard errors in parentheses, clustered at the household level; ^*^p < 0.1, ^**^p < 0.05, ^***^p < 0.01; Joint *P*-value is associated with the test of joint significance of the income shares and log gross total income; All regressions include the child fixed effects and the following time-varying controls: indicators for the survey rounds, household size, number of children under 15 and under 5, identifier for female headed household, head's education, survey month fixed effects, and age-in-months fixed effects.

We see again that increased share of income coming from crop production corresponds to lower HAZ scores. However, the coefficients for HAZ on the consumption of own crop production are and statistically significant and nearly twice the magnitude as for production. Thus, consumption of own production rather than production alone seems to drive the negative crop result in the context of Uganda. In fact, in the joint specification that includes both share of crops in income and share of consumption of own production (column 5), consumption of own crops share becomes more negative, while the crop production share changes sign and becomes small, positive, and marginally significant. While this result needs more information about other mechanisms (food choice, care patterns) that accompany a shift toward more own-consumption, it suggests the possibility that households in rural Uganda could be a little bit better off converting produce into cash for nutrition-supporting purchases, or that there may be interventions that could help households who tend to consume their own production to protect young children from any associated nutritional disadvantages.

Production of the low-protein crops shows a significant negative coefficient similar to consumption of own crop production, at 0.44 (column 3) predicted decrease from a shift from 0 to 100% plantain or cassava production, and the coefficient for consumption of own-produced low-protein crops is even more negative at −0.62 (column 4), suggesting that on average in the sample, crops may better serve long-term nutrition when converted to cash than through direct consumption. Supporting these results, column 6 combines crop share and low-protein crop share. The magnitude of low-protein crops holds but falls to marginal significance (Joint *P*-value 0.056), and the magnitude coefficient for crop share shrinks to close to zero. With crop share, low-protein production, and low-protein consumption (column 7), crop share remains small and negative, low-protein production share becomes positive, and low-protein consumption share becomes even more negative at −0.72 (though not statistically different than when included alone in column 4). For the extreme case of a child whose household’s whole income comes from production of low-protein crops, this result predicts approximately 0.5 standard deviations difference in HAZ between consuming all of that produce and selling all of it.

Underlying these results, there is a 0.78 versus 0.45 correlation between consumption and production of low-protein versus other food crops (not shown), respectively, pointing to income growth in the form of cassava and plantain production being particularly unlikely to convert into consumption of other foods or nonfoods. These anthropometric score results suggest that this “stickiness” of crop production to own consumption that may, in a context with low-protein staple crops, may potentially render agricultural growth less beneficial for nutrition than other types of growth, and that in Uganda, all else equal, there are likely to be nutritional gains from shifting toward more nutrient-rich crop production.

### (c) Joint specifications

Table 3b, Table 3c provide alternate specifications, including multiple types of shares. As discussed above, selected columns of Table 3b combines multiple types of crop shares, and Table 3c includes sector shares and crop-type shares, with wage income always as the omitted category. We see that the results with these combined shares are largely consistent with the individual specification in magnitude, though significance levels fluctuate in some specifications. Overall, it does not appear that underlying correlations between share types are driving our results.

**Table 3c:** HAZ on income shares (shares of gross sector income and child fixed effects)

Whole Sample (2325 observations in 757 clusters)			
	(1)	(2)	(3)
% Income, Crop Production	−0.166	0.0265	−0.0101
	(0.141)	(0.170)	(0.168)

% Income, Livestock	−0.0350	−0.0375	−0.0355
	(0.123)	(0.162)	(0.162)

% Income, Self-Employment	0.193	0.199	0.206
	(0.156)	(0.157)	(0.157)

% Income, Low-Protein		−0.428^*^	0.159
Crop Production		(0.232)	(0.314)
% Income, Consumption of Own Low-Protein Crop Production			−0.736^**^
			(0.345)

Ln Gross Total Income	0.00781	0.0144	0.00108
	(0.0391)	(0.0393)	(0.0402)

**Joint *P*-value**	0.111	0.0798	0.0519
**Joint *P*-value (shares)**	0.0987	0.0779	0.0541
***R*-Sq**	0.229	0.236	0.239
**Adj *R*-Sq**	0.204	0.210	0.212

*Notes:* Standard errors in parentheses, clustered at the household level; ^*^p < 0.1, ^**^p < 0.05, ^***^p < 0.01; Joint *P*-value is associated with the test of joint significance of the income shares and log gross total income; Joint *P*-value (shares) is associated with the test of joint significance of the income shares only. All regressions include the child fixed effects and the following time-varying controls: indicators for the survey rounds, household size, number of children under 15 and under 5, identifier for female headed household, head's education, survey month fixed effects, and age-in-months fixed effects.

### (d) Subsamples by age and median income

Table 4a, Table 4b, Table 4c, Table 5a, Table 5b, Table 5c present results originating from specifications comparable to those underlying the results in Table 3a, Table 3b, but this time broken down by subsamples of (i) under vs. over 24 months of age in the first year (Table 4a, Table 4b, Table 4c) and (ii) below the median income at baseline (Table 5a, Table 5b), respectively. When the results are broken down by age group, we see that the magnitudes for various crop income shares are larger and more significant for the older group (Table 4b, Table 4c)—this appears to be the group that drives the results from the full sample. This may be surprising, since the largest losses in height are expected to occur during the first years that are considered more critical for nutrition. On the other hand, we may infer that effects on the younger children may be partially mitigated by breastfeeding or by different levels of care as children pass from infancy to toddlerhood. We do see that the younger group continues to have a slightly larger magnitude for the coefficient on total income (still not significant). One possible explanation is that because a portion of the younger group is still breastfeeding, complementary feeding with low-protein foods or any kind of food contributes needed supplementary carbohydrates, while for older children, consumption of low-nutrient foods displaces more nutrient-dense consumption. Breastfeeding could also affect level of adult supervision, which may influence total amounts consumed. Given the lack of data on children’s individual consumption or care, however, it is not possible to distinguish these or other mechanisms.

**Table 4a:** HAZ on income shares (shares of gross sector income and child fixed effects)

Sub-samples by Age in First Round								
	Under 24 Months (1069 obs in 496 clusters)				24 Months and Up (1421 obs in 600 clusters)			
	(1)	(2)	(3)	(4)	(5)	(6)	(7)	(8)
% Income, Crop Production	−0.153				−0.113			
	(0.221)				(0.121)			

% Income, Livestock		−0.226				−0.0227		
		(0.218)				(0.129)		

% Income, Self-Employment			0.346^*^				0.140	
			(0.194)				(0.131)	

% Income, Wage Employment				−0.0317				−0.0361
				(0.222)				(0.111)

Ln Gross Total Income	0.0652	0.0790	0.0501	0.0878	0.0146	0.0278	0.0164	0.0295
	(0.00760)	(0.0660)	(0.0605)	(0.0728)	(0.0345)	(0.0318)	(0.0329)	(0.0327)

**Joint *P*-value**	0.269	0.216	0.154	0.340	0.418	0.681	0.370	0.666
***R*-Sq**	0.394	0.394	0.397	0.393	0.129	0.127	0.129	0.128
**Adj *R*-Sq**	0.359	0.359	0.362	0.358	0.0933	0.0.0916	0.0933	0.0917

*Notes:* Standard errors in parentheses, clustered at the household level; ^*^p < 0.1, ^**^p < 0.05, ^***^p < 0.01; Joint *P*-value is associated with the test of joint significance of the income shares and log gross total income; All regressions include the child fixed effects and the following time-varying controls: indicators for the survey rounds, household size, number of children under 15 and under 5, identifier for female headed household, head's education, survey month fixed effects, and age-in-months fixed effects. Children may appear in both subsamples if present for all three rounds and under 24 months in first appearance and at least 24 months in second.

**Table 4b:** HAZ—crop production and own consumption (shares of gross total income and child fixed effects)

Sub-samples by Age in First Round								
	Under 24 Months (1069 obs in 496 clusters)				24 Months and Up (1421 obs in 600 clusters)			
	(1)	(2)	(3)	(4)	(5)	(6)	(7)	(8)
% Income, Crop Production	−0.153				−0.113			
	(0.221)				(0.121)			

% Income, Consumption of Own Crop Production		−0.156				−0.378^**^		
		(0.303)				(0.176)		

% Income, Low-Protein Crop Production			−0.320				−0.389^**^	
			(0.313)				(0.184)	

% Income, Consumption of Own Low-Protein Crop Production				−0.368				−0.615^***^
				(0.350)				(0.210)

Ln Gross Total Income	0.0652	0.0683	0.0721	0.0669	0.0146	−0.00966	0.0141	0.00535
	(0.00760)	(0.0810)	(0.0676)	(0.0694)	(0.0345)	(0.0383)	(0.0317)	(0.0324)

**Joint *P*-value**	0.269	0.280	0.224	0.206	0.418	0.0479	0.0653	0.00751
***R*-Sq**	0.394	0.393	0.395	0.395	0.129	0.134	0.134	0.0138
**Adj *R*-Sq**	0.359	0.359	0.360	0.360	0.093	0.0989	0.0982	0.103

*Notes:* Standard errors in parentheses, clustered at the household level; ^*^p < 0.1, ^**^p < 0.05, ^***^p < 0.01; Joint *P*-value is associated with the test of joint significance of the income shares and log gross total income; All regressions include the child fixed effects and the following time-varying controls: indicators for the survey rounds, household size, number of children under 15 and under 5, identifier for female headed household, head's education, survey month fixed effects, and age-in-months fixed effects.

**Table 4c:** HAZ—crop production and own consumption (shares of gross total income and child fixed effects)

Sub-samples by Age in First Round								
	Under 24 Months (1069 obs in 496 clusters)				24 Months and Up (1421obs in 600 clusters)			
	(1)	(2)	(3)	(4)	(5)	(6)	(7)	(8)
% Income, Crop Production	−0.133	0.0215	−0.00137	−0.138	0.211	0.0935	−0.0675	0.198
	(0.291)	(0.249)	(0.253)	(0.314)	(0.211)	(0.148)	(0.147)	(0.213)

% Income, Consumption of Own Crop Production	−0.0312			0.330	−0.594^**^			−0.316
	(0.414)			(0.435)	(0.299)			(0.340)

% Income, Low-Protein Crop Production		−0.338	−0.0966	−0.149		−0.479^**^	0.0598	0.0345
		(0.366)	(0.415)	(0.417)		(0.224)	(0.414)	(0.409)

% Income, Consumption of Own Low-Protein Crop Production			−0.277	−0.397			−0.735^*^	−0.543
			(0.417)	(0.424)			(0.437)	(0.434)

Ln Gross Total Income	0.0643	0.0743	0.0673	0.0777	0.00786	0.0213	0.0104	0.0000
	(0.0806)	(0.0762)	(0.0796)	(0.0847)	(0.0380)	(0.0341)	(0.0351)	(0.0387)

**Joint *P*-value**	0.445	0.391	0.522	0.726	0.0766	0.112	0.522	0.0640
***R*-Sq**	0.394	0.395	0.395	0.396	0.136	0.134	0.139	0.140
**Adj *R*-Sq**	0.358	0.359	0.359	0.395	0.100	0.0980	0.102	0.103

*Notes:* Standard errors in parentheses, clustered at the household level; ^*^p < 0.1, ^**^p < 0.05, ^***^p < 0.01; Joint *P*-value is associated with the test of joint significance of the income shares and log gross total income; All regressions include the child fixed effects and the following time-varying controls: indicators for the survey rounds, household size, number of children under 15 and under 5, identifier for female headed household, head's education, survey month fixed effects, and age-in-months fixed effects.

**Table 5a:** HAZ on income shares (shares of gross sector income and child fixed effects)

Sub-sample: Below Median Income in First Year (1574 obs in 577 clusters)				
	(1)	(2)	(3)	(4)
% Income, Crop Production	−0.101			
	(0.133)			

% Income, Livestock		−0.151		
		(0.150)		

% Income, Self-Employment			0.00294	
			(0.136)	

% Income, Wage Employment				0.275^**^
				(0.131)

Ln Gross Total Income	0.0226	0.0356	0.0371	0.0181
	(0.0417)	(0.0365)	(0.0375)	(0.0381)

**Joint *P*-value**	0.420	0.371	0.588	0.0532
***R*-Sq**	0.311	0.311	0.310	0.313
**Adj *R*-Sq**	0.276	0.277	0.276	0.279

*Notes:* Standard errors in parentheses, clustered at the household level; ^*^p < 0.1, ^**^p < 0.05, ^***^p < 0.01; Joint *P*-value is associated with the test of joint significance of the income shares and log gross total income; All regressions include the child fixed effects and the following time-varying controls: indicators for the survey rounds, household size, number of children under 15 and under 5, identifier for female headed household, head's education, survey month fixed effects, and age in months fixed effect.

**Table 5b:** HAZ—crop production and own consumption (shares of gross total income and child fixed effects)

Sub-sample: Below Median Income in First Year (1574 obs in 577 clusters)				
	(1)	(2)	(3)	(4)
% Income, Crop Production	−0.101			
	(0.133)			

% Income, Consumption of Own Crop Production		−0.338^**^		
		(0.189)		

% Income, Low-Protein Crop Production			−0.211	
			(0.184)	

% Income, Consumption of Own Low-Protein Crop Production				−0.338^*^
				(0.182)

Ln Gross Total Income	0.0226	0.00367	0.0295	0.0226
	(0.0417)	(0.0433)	(0.0372)	(0.0376)

**Joint *P*-value**	0.420	0.103	0.291	0.131
***R*-Sq**	0.311	0.314	0.311	0.313
**Adj *R*-Sq**	0.276	0.280	0.277	0.279

*Notes:* Standard errors in parentheses, clustered at the household level; ^*^p < 0.1, ^**^p < 0.05, ^***^p < 0.01; Joint *P*-value is associated with the test of joint significance of the income shares and log gross total income; All regressions include the child fixed effects and the following time-varying controls: indicators for the survey rounds, household size, number of children under 15 and under 5, identifier for female headed household, head's education, survey month fixed effects, and age-in-months fixed effects.

**Table 5c:** HAZ—crop production and own consumption (shares of gross total income and child fixed effects)

Sub-sample: below median income in the first year (1,574 obs in 577 clusters)				
	(1)	(2)	(3)	(4)
% Income, Crop Production	0.271	−0.0046	−0.0396	0.187
	(0.209)	(0.156)	(0.158)	(0.225)
% Income, Consumption of Own Crop Production	−0.603^*^			−0.507
	(0.296)			(0.352)

% Income, Low-Protein		−0.207	0.456	0.457
Crop Production		(0.218)	(0.343)	(0.332)

% Income, Consumption of			−0.790^**^	−0.545
Own Low-Protein Crop Production			(0.373)	(0.386)

Ln Gross Total Income	0.00353	0.0290	0.0168	−0.0018
	(0.0433)	(0.0418)	(0.0426)	(0.0446)

**Joint *P*-value**	0.131	0.480	0.204	0.171
***R*-Sq**	0.315	0.311	0.314	0.317
**Adj *R*-Sq**	0.281	0.277	0.279	0.281

*Notes:* Standard errors in parentheses, clustered at the household level; ^*^p < 0.1, ^**^p < 0.05, ^***^p < 0.01; Joint *P*-value is associated with the test of joint significance of the income shares and log gross total income; All regressions include the child fixed effects and the following time-varying controls: indicators for the survey rounds, household size, number of children under 15 and under 5, identifier for female headed household, head's education, survey month fixed effects, and age-in-months fixed effects.

For children whose household income was below the median value in the first year, the results generally follow the overall sample results with a few differences. Across sectors, there appears to be little premium for self-employment but a larger gain in wage labor. We speculate that for poorer households, self-employment may more often be an option of last resort or may require costly inputs to be more profitable, and wage income may provide a more available or more predictable income during lean periods. For crops, the “penalty” for own consumption, especially of the low-protein crops, appears to grow in magnitude relative to production for sale. It is difficult to say without more information, but it is possible that this subset of children has a less nutritious diet at the start, such that marginal food consumption could be even more important.

## 6 Conclusion

In this study, we have used panel data to explore the potential for short-term income gains to improve children’s nutrition in Uganda. The high frequency in the UNPS—three rounds in three years—allow for fixed effect estimations that eliminate time-invariant unobservable factors that may typically confound similar studies. Without convincing instrumental variables for income, one still must take care with causal interpretations, but such exercises can serve as informative diagnostic tools for policy and future research.

Our results show very little relationship between changes in log income levels and height *z*-scores, considered a good marker of long-term nutrition. Contrary to the benchmark case of in which income generation might be uniformly good for nutrition, however, increased self-employment income appears to correlate relatively more with positive nutritional outcomes than other sectors. Future studies distinguishing the mechanisms behind this trend, potentially dietary or related to proximity to home for childcare, would be worthwhile.

Specific to Uganda, we find the potentially less expected result that agricultural income appears to be more nutrition-negative than others, seemingly through the production of low-nutrient crops and specifically through own consumption. Our results suggest the possibility of stickiness of crop production to own consumption; while this may be a nutrition-supporting feature in other contexts, income growth in the form of production of low-nutrient crops may crowd out consumption of other goods and services that could serve as better nutritional investments. These results appear to be concentrated among the older and poorer subset of children in our sample. Still, any effects appear to be relatively small in magnitude, especially given that year-on-year changes in income for households usually fall within limited bounds. These results are also likely to depend heavily on the agricultural and dietary profile of Uganda and caution against uniform policies to support one sector over another without further information specifically about how children’s diets or childcare patterns accompany income changes. When data are available, similar diagnostic techniques may be useful for identifying which sectors and interventions may be most nutrition-supporting in other contexts, but would be best complemented with more details about household practices for child feeding and care.

## Footnotes

1 Some examples include (i) the International Food Policy Research Institute (IFPRI) “2020 Conference: Leveraging Agriculture for Improving Nutrition and Health” that was held in New Delhi, India in February 2011, and (ii) the United States Agency for International Development (USAID)’s review of nutrition and food security impacts of agriculture projects, which can be found here: http://pdf.usaid.gov/pdf_docs/PNADY253.pdf.

2 Masset *et al.* (2011) narrow down their focus on interventions with an explicit goal of improved child nutrition. The 2011 online version of the publication offer additional details on counterfactual analysis, power, intermediate outcomes, and heterogeneity of impacts, and can be found here: http://r4d.dfid.gov.uk/PDF/Outputs/SystematicReviews/Masset_etal_agriculture_and_nutrition.pdf.

3 While “agriculture” can refer to both crop cultivation and animal rearing, we focus primarily on crops and not animals.

4 There is a large literature establishing the importance of all three factors for nutritional outcomes, which we take as given, though a precise nutrition production function is widely absent, given measurement difficulties and identification challenges that arise from reliance on observational data.

5 Given the limitations of nutritional science to define the biological relationships more precisely, and acknowledging the admonitions of Behrman and Deolalikar (1988), we do not detail a functional form of utility on health or of health on the relevant inputs. We will rely on multiple specifications and robustness checks rather than claiming a structural form.

6 Headey (2013) is able to point to greater food production, though not consumption in particular.

7 See Timmer (1997) and Strasberg, Jayne, Yamano, Nyoro, Karanja, and Strauss (1999) for a general discussion and a discussion on Kenya, respectively.

8 Many interventions also incorporate educational components to try to increase preference for nutrition-supporting consumption, a mechanism outside of what we can test using the current dataset.

9 It is unclear whether these changes arise through agricultural growth among the poor or through falling food prices economy-wide.

10 The UNPS data and documentation are publically available on www.worldbank.org/lsms.

11 Income measures have been constructed following the cross-country comparable Rural Income Generating Activities (RIGA) income aggregate methodology. More information on RIGA is available on http://www.fao.org/economic/riga/rural-income-generating-activities/en/.

12 The former asks about consumed quantities for the year out of production totals, the latter asks about quantities from own production for the past week out of consumption totals.

13 In each wave, the UNPS collected information on smallholder agriculture for the first and second seasons of a specific calendar year. Each season spans a period of 6 months; the first season lasts from January to June, while the second season lasts from July to December. In 2009–10, 2010–11, and 2011–12 waves, the UNPS collected information on the first and second seasons of 2009, 2010, and 2011, respectively. In each wave, the first season specific information was collected in Visit 1, while the second-season-specific information was collected in Visit 2. Visit 1 and Visit 2 fieldwork each spanned a period of 6 months. In a given visit, the household was visited well after the reference season of interested was concluded; between 3 to 9 months in 2009–10, 4 to 10 months in 2010–11 and 5 to 11 months in 2011–12. This delay increases the probability that consumption from own production would be “complete” at the time of the interview. The specific question for the quantification of own production that is consumed at home in a given season is asked at the parcel-plot-crop-condition-level in Section 5 of the agriculture questionnaire, and states “How much of the […] harvested during the first season of 2013 has already been consumed by your household?” Finally, our regressions do control for the month of visit 1 interview fixed effects in each wave, which in part pick up the differential cognitive demands of recall across the household sample as well as any other seasonal effects.

14 Livestock income is the exception to this. Due to changes in the livestock modules of the agriculture questionnaire during the three waves of the UNPS, the estimated value of livestock and livestock by-product production consumed at home is derived from the food consumption section of the household questionnaire.

15 Anecdotally, there is also some concern that the livestock modules may have suffered differential attrition relative to other modules over the course of the panel series, perhaps related to survey fatigue and the fact that the livestock modules were administered after several other modules.

16 The out-of-sample population additionally includes (i) children whose birthdates did not match between survey rounds and could not be reconciled using reported age in months, (ii) a small number of observations who were missing household income data, and (iii) eight children who change households within the UNPS sample across survey rounds.

17 We assume that child and time fixed effects as well time variant observables included in our regressions, as detailed in Section 4, including household size, number of children under 15 and under 5, identifier for female headed household, head's education, survey month fixed effects, and child age-in-months fixed effects, capture any remaining unobserved heterogeneity that might jointly predict the likelihood of being out-of-sample and the outcome variables of interests. This assumption is empirical untestable and is equivalent to the assumption of selection on observables.

18 We choose to focus on gross income for a number of reasons, including differences in the ways that expenses were collected across years, small numbers of households reporting any expenditure, and the difficulty of shares in the presence of negative numbers. Gross income may also better represent intensity of income activity by sector.

19 According to WHO guidelines, we exclude children whose scores fall outside recommended cutoffs for plausible values: HAZ below −6 or above +6, WAZ below −6 or +5, WHZ below −5 or above +5. Source: http://www.who.int/nutgrowthdb/software/Differences_NCHS_WHO.pdf.

20 In all reported results, HAZ was calculated using reported date of birth when birth date and reported age in months did not correspond. HAZ using reported age in months generally yields similar results.

21 Child age has been calculated taking into account the date of birth and the interview date, rather than the reported age in months, when these two measures did not match in the data.

22 A random effects specification was tested against the fixed effects specification, but was rejected in favor of the fixed effects with a Hausman test chi-2 p-value of smaller than 0.0001.

23 Shares were chosen to prevent over-weighting of large-income households, who are likely to have larger nominal year-on-year fluctuations of reported income. Typically, logs would be used for this purpose, but shares allow us to keep households who do not have positive values for all income categories, where they are undefined as log(0).

24 An alternative estimation strategy that was beyond the scope of this paper would be to include per capita gross income from each of the sectors simultaneously.

25 We started with three categories: (1) low-protein food crops, (2) other food crops, and (3) non-food crops, but found too few observations and too little variation for non-food crops to treat them separately.

26 While plantains are a source of Vitamin A and other micro-nutrients, they are particularly low in protein, a necessary component for growth and development and most biological processes. Plantain and cassava have only a fraction of the protein content in cereals, which are also considered limited in their ability to meet preschoolers’ protein needs without complementary higher protein foods. (FAO (1997)—http://www.fao.org/docrep/w0078e/w0078e08.htm).
